# Characterization of chromatin accessibility patterns in different mouse cell types using machine learning methods at single-cell resolution

**DOI:** 10.3389/fgene.2023.1145647

**Published:** 2023-03-01

**Authors:** Yaochen Xu, FeiMing Huang, Wei Guo, KaiYan Feng, Lin Zhu, Zhenbing Zeng, Tao Huang, Yu-Dong Cai

**Affiliations:** ^1^ Department of Mathematics, School of Sciences, Shanghai University, Shanghai, China; ^2^ School of Life Sciences, Shanghai University, Shanghai, China; ^3^ Key Laboratory of Stem Cell Biology, Shanghai Jiao Tong University School of Medicine (SJTUSM) and Shanghai Institutes for Biological Sciences (SIBS), Chinese Academy of Sciences (CAS), Shanghai, China; ^4^ Department of Computer Science, Guangdong AIB Polytechnic College, Guangzhou, China; ^5^ School of Health Science and Engineering, University of Shanghai for Science and Technology, Shanghai, China; ^6^ Bio-Med Big Data Center, CAS Key Laboratory of Computational Biology, Shanghai Institute of Nutrition and Health, Chinese Academy of Sciences, University of Chinese Academy of Sciences, Shanghai, China; ^7^ CAS Key Laboratory of Tissue Microenvironment and Tumor, Shanghai Institute of Nutrition and Health, Chinese Academy of Sciences, University of Chinese Academy of Sciences, Shanghai, China

**Keywords:** chromatin accessibility, chromatin heterogeneity, single-cell resolution, mouse cell type, machine learning, biomarker genes

## Abstract

Chromatin accessibility is a generic property of the eukaryotic genome, which refers to the degree of physical compaction of chromatin. Recent studies have shown that chromatin accessibility is cell type dependent, indicating chromatin heterogeneity across cell lines and tissues. The identification of markers used to distinguish cell types at the chromosome level is important to understand cell function and classify cell types. In the present study, we investigated transcriptionally active chromosome segments identified by sci-ATAC-seq at single-cell resolution, including 69,015 cells belonging to 77 different cell types. Each cell was represented by existence status on 20,783 genes that were obtained from 436,206 active chromosome segments. The gene features were deeply analyzed by Boruta, resulting in 3897 genes, which were ranked in a list by Monte Carlo feature selection. Such list was further analyzed by incremental feature selection (IFS) method, yielding essential genes, classification rules and an efficient random forest (RF) classifier. To improve the performance of the optimal RF classifier, its features were further processed by autoencoder, light gradient boosting machine and IFS method. The final RF classifier with MCC of 0.838 was constructed. Some marker genes such as *H2-Dmb2*, which are specifically expressed in antigen-presenting cells (e.g., dendritic cells or macrophages), and *Tenm2*, which are specifically expressed in T cells, were identified in this study. Our analysis revealed numerous potential epigenetic modification patterns that are unique to particular cell types, thereby advancing knowledge of the critical functions of chromatin accessibility in cell processes.

## 1 Introduction

Chromatin accessibility is a generic property of the eukaryotic genome, which refers to the degree of physical compaction of chromatin (Klemm et al., 2019). Chromatin is a complex of DNA and associated proteins that form chromosomes and present varied states across genomes, tissues, and cell types (Lee et al., 2004). Nucleosome occupancy is variably dynamic, indicating that densely arranged nucleosomes lead to closed chromatin, whereas partially depleted nucleosomes result in accessible or permissive chromatin (Lee et al., 2004; Poirier et al., 2008; Sheffield and Furey, 2012; Klemm et al., 2019). Evidence demonstrates that nucleosomes are typically depleted at the transcriptional regulatory region, including enhancers, promoters, and other transcription factor binding loci (Ozsolak et al., 2007; Thurman et al., 2012). The distinct chromatin accessibility patterns directly reflect different functional states, and they are modulated through a variety of mechanisms, such as histone methylation, acetylation, and DNA methylation (Allis and Jenuwein, 2016). These modifications change the interplays between transcriptional regulators and DNA targets, thereby altering the downstream gene expressions and affecting cell functions. Various changes in chromatin structure and modification have been involved in a range of traits and diseases (Hendrich and Bickmore, 2001). Therefore, characterizing the chromatin accessibility is a critical demand for understanding their functional roles in gene regulation during development and in disease contexts.

In general, the measurement of chromatin accessibility is dependent on the physical access of enzymes to target fragments. Hewish et al. first noticed the periodic hypersensitivity of chromatin to DNA endonucleases across the genome, indicating the accessible regions among nucleosomes (Hewish and Burgoyne, 1973). Combine with next-generation sequencing techniques, a genome-wide profiling of chromatin accessibility was carried out, which was known as DNase I hypersensitive site sequencing (DNase-seq) (Boyle et al., 2008). An alternative assay, namely, ATAC-seq, can profile chromatin accessibility based on Tn5 transposon (Buenrostro et al., 2013). ATAC-seq shows a higher sensitivity on low-input samples, and the protocol is less complex compared with DNase-seq. Therefore, this approach is commonly used in recent research to generate chromatin accessibility profiles.

Chromatin accessibility is cell type dependent, indicating the chromatin heterogeneity across cell lines and tissues (Thurman et al., 2012). Previous studies with bulk chromatin accessibility profiles usually attempt to obtain homogeneous cell samples to avoid bias derived from cell heterogeneity. Recently, single-cell epigenomic assays emerged and provided a new way to investigate the regulatory mechanism of chromatin accessibility in complex tissues. However, accurate cell type annotation in single-cell ATAC-seq data remains a great challenge. Thus, three main strategies of cell type annotation in single-cell ATAC-seq data were implemented, including annotation using cis-regulatory elements, annotation using cell type-specific feature set, and annotation using RNA sequencing data as reference (Corces et al., 2016; Schep et al., 2017; Pliner et al., 2018; Stuart et al., 2019). These strategies show certain limitations that either rely on reliable cell type markers or require additional reference datasets. A combinatorial indexing assay, namely, sci-ATAC-seq, was applied to profile the genome-wide chromatin accessibility in single cells from different mouse tissues (Cusanovich et al., 2018a). Based on these data, the heterogeneity in chromatin accessibility within cell types was characterized, and candidate tissue-specific patterns of chromatin accessibility were identified. Considering that a relatively traditional workflow was applied for analysis and only a few epigenetic markers had been found, several potential characteristic patterns of chromatin accessibility across cell types remain undiscovered.

In this study, based on the single-cell chromatin accessibility data from the atlas (Cusanovich et al., 2018a), we applied several machine learning methods to identify relevant characteristic chromatin accessibility patterns that can serve as cell-type-specific markers. The Boruta (Kursa and Rudnicki, 2010) and Monte Carlo Feature Selection (MCFS) (Micha et al., 2008) were applied to the data one by one, yielding a list containing 3897 genes. Then, the list was subjected to incremental feature selection (IFS) (Liu and Setiono, 1998) method, containing decision tree (DT) (Safavian and Landgrebe, 1991) and random forest (RF) (Breiman, 2001). IFS with RF can help to construct an efficient classifier, whereas IFS with DT was used to generate classification rules, which represent the quantitative characteristics of chromatin accessibility for distinguishing different cell types. Features used in the optimal RF classifier were further processed by autoencoder, light gradient boosting machine (LightGBM) (Ke et al., 2017) and IFS method for accessing a better classifier. The final analysis was focused on top features in the list and classification rules, confirming some potential epigenetic modification patterns in particular cell types. This study gave an important contribution to a comprehensive understanding of the essential roles of chromatin accessibility in cell functions.

## 2 Materials and methods

### 2.1 Data

Large-scale sci-ATAC-seq data were accessed from the GEO database under accession number of GSE111586 provided by Cusanovich et al. (Cusanovich et al., 2018b). The sci-ATAC-seq data were collected on 77 different cell types from 13 different tissues that contained 69,015 cells, and 77 different cell types were used as classification targets in our research. The number of cells contained in each cell type is shown in Supplementary Table S1. A total of 436,206 chromosome segments mapped to 20,783 genes were obtained by sci-ATAC-seq, and these genes and their existence status (one for existence and 0 for non-existence) in each cell were used as features in this study. Using this quantitative representation, we converted enriched chromosome segments into biologically interpretable genes, thereby providing comprehensive understanding of the classification process.

### 2.2 Boruta

The Boruta algorithm is a feature selection wrapper that can be used to any classification method that generates a variable significance measure (Kursa and Rudnicki, 2010). Boruta searches for all features that contain relevant information that may be utilized for prediction rather than concentrating on finding a restricted group of features with the lowest classification error. The Boruta algorithm consists of the following steps: 1) For each explanatory variable, a shadow variable is made, and its association with the target variable is eliminated by randomly rearranging its values. 2) RFs are built to fit the expanded data. 3) An accuracy loss z-score is applied to each variable including the original and shadow variables. 4) The original attributes are selected if their z-scores are significantly higher than those of shadow counterparts. The process is repeated until all features have been accepted or disregarded. The z-score of the original attributes must be statistically and significantly higher than the maximum z-score of the shadow attributes to identify the most pertinent features of the original attributes.

In this study, we opted for the Boruta program from https://github.com/scikit-learn-contrib/boruta_py and selected the default parameters for subsequent analysis.

### 2.3 Feature ranking algorithms

#### 2.3.1 Monte carlo feature selection

Monte Carlo feature selection is a DT-based feature importance evaluation algorithm commonly used to process biological data (Micha et al., 2008; Chen X. et al., 2019; Li et al., 2020). In MCFS, m features were randomly selected. Based on these features, *t* DTs are built with *t* randomly selected sample sets. Above procedure is repeated s times. Finally, s×t DTs were constructed. The relative importance (RI) of a feature, as measured by how many times it has been selected by these trees and how much it contributes to predicting the class of these trees, is estimated as follows:

$${RI}_{g}=\overset{st}{\underset{\tau =1}{\sum }}{\left(wAcc\right)}^{u}\underset{n_{g}\left(\tau \right)}{\sum }IG\left(n_{g}\left(\tau \right)\right){\left(\frac{no.in\;n_{g}\left(\tau \right)}{no.in\;\tau }\right)}^{v}$$ (1)

where wAcc is the weighted accuracy, $IG\left(n_{g}\left(\tau \right)\right)$ is the information gain (IG) of node $n_{g}\left(\tau \right)$ , ( $\left.no.in\;n_{g}\left(\tau \right)\right)$ is the number of samples in node $n_{g}\left(\tau \right)$ , and $\left(no.in\;\tau \right)$ is the sample size in the tree root. In addition, u and v are two settled positive integers. After each feature is assigned a RI score, all features are ranked in a list with the decreasing order of their RI scores.

This study adopted the MCFS program sourced from http://www.ipipan.eu/staff/m.draminski/mcfs.html. It was executed using its default parameters.

#### 2.3.2 Light gradient boosting machine

The LightGBM is deemed as a strong machine learning algorithm that combines several weak DTs (Ke et al., 2017). It improves the gradient boosting decision tree (GBDT) by increasing the efficiency and reducing memory usage. According to the constructed DTs, LightGBM can also be used to evaluate the importance of features. If *K* DTs are constructed, the total number of times, denoted by T Split, for each feature is computed, which is defined as the overall used times in all DTs, i.e.,

$$T\;Split=\overset{K}{\underset{i=1}{\sum }}{Split}_{i}$$ (2)

where ${Split}_{i}$ is the used times of this feature in the *i*th DT. Evidently, if T Split for one feature is large, i.e., it occurs in lots of DTs, this feature is quite important. Thus, LightGBM sorts all features in a list with the decreasing order of their T Split values.

In the present study, we utilized the LightGBM program sourced from https://lightgbm.readthedocs.io/en/latest/and ran the analysis by using the default settings.

### 2.4 Incremental feature selection

It is still quite difficult to extract essential features from a feature list to comprise an optimal feature space for a given classification algorithm. Here, we introduced IFS, a well-liked method for determining the optimal feature space (Liu and Setiono, 1998; Chen L. et al., 2019; Zhang et al., 2020; Huang et al., 2023a; Huang et al., 2023b). The main steps of IFS are as follows: 1) From the feature list, lots of feature subsets are constructed with a fixed step, each of which contained some top features in the list. 2) One classifier is built on each constructed feature subset with a given classification algorithm and it is evaluated by 10-fold cross-validation (Kohavi, 1995). 3) The classifier with the best classification performance is selected as the optimal classifier and features used in this classifier are referred as the optimal features.

### 2.5 Synthetic minority oversampling technique

Among the 77 cell types, a 70-fold difference was observed between the largest number of cells and the smallest number of cells. It was not easy to build a fair classifier on such imbalanced dataset. The SMOTE is a data augmentation technique, which can be used to balance out the imbalanced dataset (Chawla et al., 2002). It tackles the imbalanced problem by employing new samples to minority classes. In particular, a sample is randomly selected from each minority class. Then, k closest neighbors of this sample in the same class are picked up and one neighbor is randomly selected. With this sample and its randomly selected neighbor, a synthetic sample is constructed at a randomly selected location in the feature space between them. In this study, the SMOTE algorithm was implemented *via* Python. Each class except the largest class was processed by SMOTE so that it contained the same number of samples in the largest class.

### 2.6 Classification algorithm

Classification algorithm is necessary for IFS method. Here, two algorithms were used: DT (Safavian and Landgrebe, 1991) and RF (Breiman, 2001). Their brief introduction is as below.

#### 2.6.1 Decision tree

DT is a basic classification and regression method with tree-like structures (Safavian and Landgrebe, 1991; Zhang et al., 2021). A DT model represents the classification and discrimination of data as a tree-like structure with nodes and directed edges. Based on one path of a DT from the root node to the leaf node, a rule can be set up, where each internal node corresponds to the rule’s condition, and a leaf node displays the outcome of an associated rule. Thus, a collection of if–then rules can be extracted from a DT. In implementing DT, we used the CART method and the scikit-learn package, with Gini coefficients serving as the IG (Pedregosa et al., 2011).

#### 2.6.2 Random forest

RF is an ensemble method, and its basic unit is DT (Breiman, 2001; Li et al., 2022; Ran et al., 2022; Yang and Chen, 2022; Wang and Chen 2023). Each DT was created based on randomly selected features and samples. For a given test sample, each tree provides its prediction. RF integrates these predictions using majority voting. In this study, the RF package from Python’s scikit-learn module was used for constructing RF classifiers.

### 2.7 Autoencoder

Autoencoders are a type of deep learning algorithm that are very useful in the field of unsupervised learning (Hinton and Salakhutdinov, 2006; LeCun et al., 2015). They are a specific type of feedforward neural networks that are designed to receive an input and transform it into a different representation, which compress the data and reduce its dimensionality. Autoencoders compress the input into a lower-dimensional embedding and then reconstruct the output from this embedding, which is a lower-dimensional representation for a higher-dimensional data.

Autoencoders consist of three modules: encoder, embedding and decoder. The encoder maps the input data into the embedding. The embedding contains the compressed knowledge representation, which is typically smaller than the input data. The decoder reconstructs the input data back from the embedding. Autoencoder networks would perform as close to the perfect reconstruction as possible.

Assume we have an input data ***x*** with *d*-dimension, autoencoders first learn a mapping from ***x*** to ***y***.

$$y=f\left(Wx+b\right)$$ (3)

where *f* is a non-linear function. After this mapping is done, autoencoders learn a mapping from the embedding ***y*** back to reconstruction ***z*** of the same shape as ***x***, which can be expressed as:

$$z=g\left(W^{T}y+b^{\prime }\right)$$ (4)

where *g* is another non-linear function. The loss function used to train autoencoders is called reconstruction loss, which is typically measured using MSE Loss or L1 Loss between ***x*** and ***z***.

$$L=\left\|x-z\right\|$$ (5)

where ***z*** represents the predicted output and ***x*** represents the input data.

The reconstruction loss can be minimized by any mathematical optimization technique, but usually be accomplished by stochastic gradient descent (SGD) (Le, 2013). ***Z*** can be used as the low-dimensional embeddings of the samples.

In this study, autoencoder was used to process the optimal features obtained by IFS method. The reconstructed features were evaluated by LightGBM and the generated list was fed into IFS method again to set up a more efficient classifier.

### 2.8 Performance evaluation

The MCC is a comparatively balanced indicator that can be applied when the sample size is unbalanced. The range of MCC is [−1, 1], where a value of one indicates that predictions and actual results match up perfectly; a value of 0 indicates that the predictions are like random predictions, and −1 indicates that the actual outcomes differ from the prediction in a negative way. Thus, MCC can describe the strength of the correlation between the expected and actual results. For the multi-class classification problem, MCC can be calculated by using the following formula (Gorodkin, 2004; Liu et al., 2021; Pan et al., 2022; Tang and Chen, 2022; Wang and Chen, 2022; Zhang et al., 2022; Wu and Chen, 2023):

$$MCC=\frac{cov\left(X,Y\right)}{\sqrt{cov\left(X,X\right)cov\left(Y,Y\right)}}=\frac{\frac{1}{K}\overset{N}{\underset{n=1}{\sum }}\overset{K}{\underset{k=1}{\sum }}\left(X_{nk}-{\bar{X}}_{k}\right)\left(Y_{nk}-{\bar{Y}}_{k}\right)}{\sqrt{\overset{N}{\underset{n=1}{\sum }}\overset{K}{\underset{k=1}{\sum }}{\left(X_{nk}-{\bar{X}}_{k}\right)}^{2}\overset{N}{\underset{n=1}{\sum }}\overset{K}{\underset{k=1}{\sum }}{\left(Y_{nk}-{\bar{Y}}_{k}\right)}^{2}}},$$ (6)

where *N* is the number of samples, *K* denotes the number of classes, X is the binary matrix into which the predicted class of each sample is converted by one-hot encoding; Y is the binary matrix into which the true class of each sample is converted by one-hot encoding, and $cov\left(X,Y\right)$ is the covariance of two matrices. ${\bar{X}}_{k}$ and ${\bar{Y}}_{k}$ are the means of the k - th column of matrices X and Y , respectively. $X_{nk}$ and $Y_{nk}$ are the elements in the n - th row and k - th column of the matrices X and Y , respectively. In this study, MCC was adopted as the major measurement to assess the performance of classifiers.

In addition, we also employed other two measurements: individual accuracy and overall accuracy (ACC). Individual accuracy indicates the prediction quality of the classifier on one class, which is defined as the proportion of correctly predicted samples in this class. ACC represents the overall performance of the classifier. It is defined as the proportion of correctly predicted samples to all samples.

## 3 Results

**FIGURE 1:**
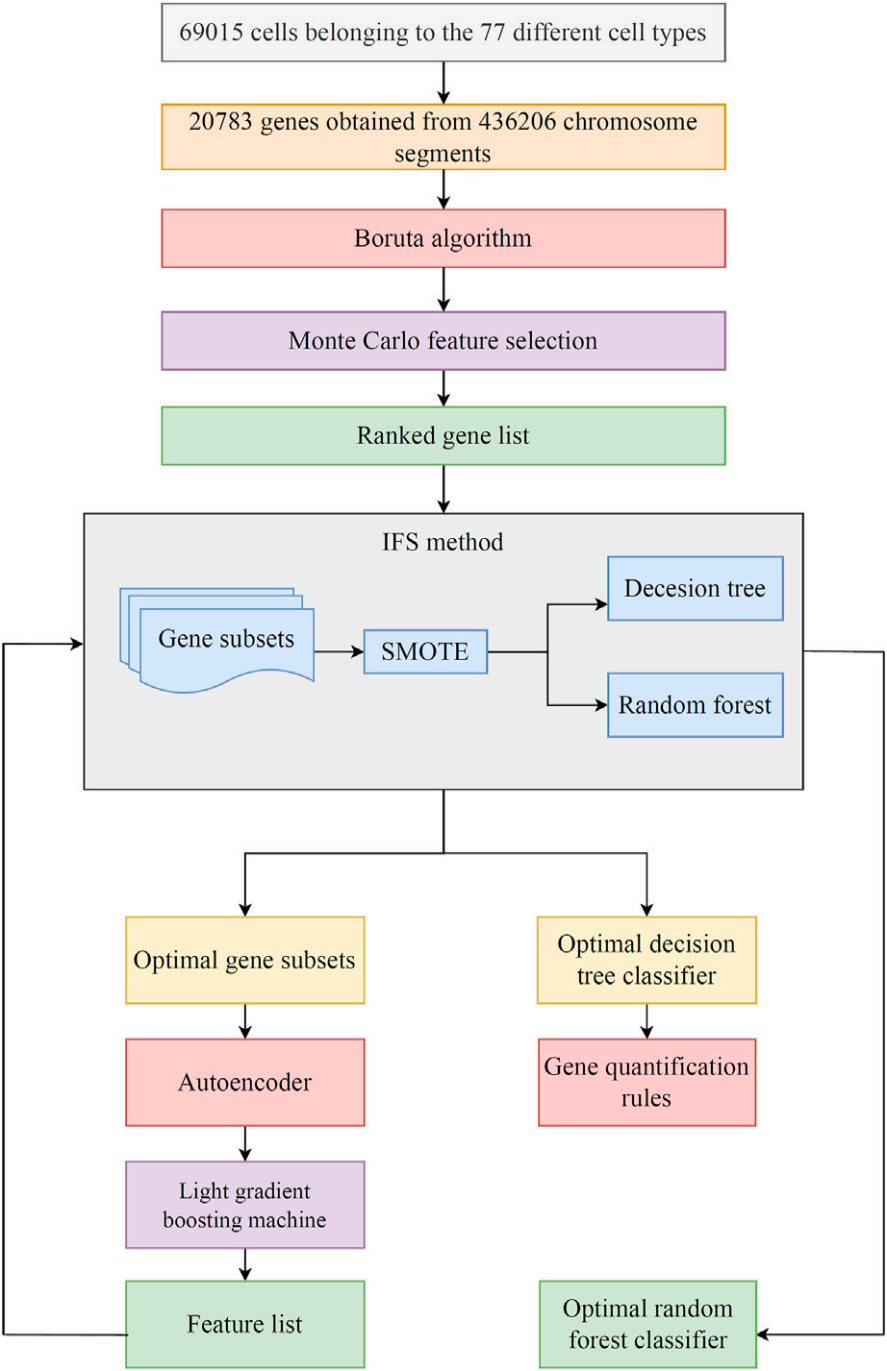
Flowchart of the machine learning procedure in this study. The transcriptionally active chromosome segments are identified by sci-ATAC-seq at single-cell resolution, including 69,015 cells belonging to 77 different cell types and 436,206 active chromosome segments mapped to 20,783 genes. The Boruta algorithm is used to filter genes, and then genes are ranked in accordance with the Monte Carlo feature selection algorithm. Subsequently, the optimal classifier and corresponding optimal feature subsets are obtained using incremental feature selection (IFS) and two classification algorithms. The classification rules are mined by the optimal decision tree (DT) classifier. Finally, the optimal features for random forest (RF) are reconstructed by autoencoder. The reconstructed features are evaluated by LightGBM, resulting in a feature list. IFS method is applied on such list to set up the final optimal RF classifier.

In the current work, we used efficient feature selection methods and classification algorithms to mine significant features in various cell types to identify relevant characteristic chromatin accessibility patterns that can serve as cell-type-specific markers. Figure 1 displays the overall analysis framework. The description of the outcomes connected to each step was listed in this section.

### 3.1 Feature ranking results

The current study included 77 cell types with a total of 69,015 and 20,783 genes. The gene features were first analyzed by Boruta. 3897 features were selected by Boruta, which are provided in Supplementary Table S2. Then, these features were investigated by MCFS, resulting in a feature list. Such list is also provided in Supplementary Table S2. The list would be entered into the IFS approach to determine the optimal features for constructing the optimal classifiers.

### 3.2 Results of IFS with RF and DT algorithms

**FIGURE 2:**
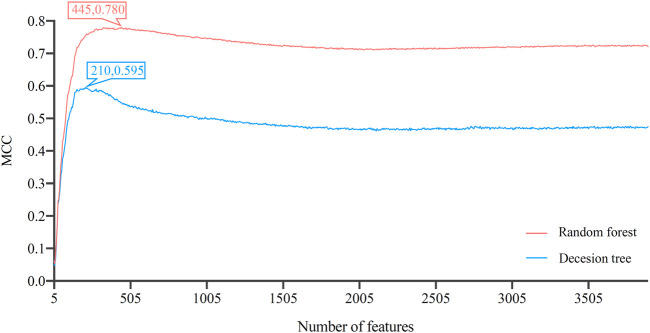
Incremental feature selection (IFS) curves of decision tree and random forest. The optimal classification performance alone with the optimal feature number for each algorithm has been labeled on the curve. Random forest has better classification results than decision tree.

After the Boruta and MCFS feature selecting methods, 3897 genes were sorted in a list. Such list was then partitioned into 779 feature subsets by five-step intervals in IFS method. On each feature subset, one DT classifier and one RF classifier were built. Their performance was evaluated through 10-fold cross-validation. As mentioned in Section 2.8, MCC was selected as the major measurement. The IFS curves, as shown in Figure 2 **,** for the two classification algorithms were plotted, where MCC and number of features were set as the *Y*-axis and *X*-axis, respectively. The detailed results of IFS are provided in Supplementary Table S3.

**FIGURE 3:**
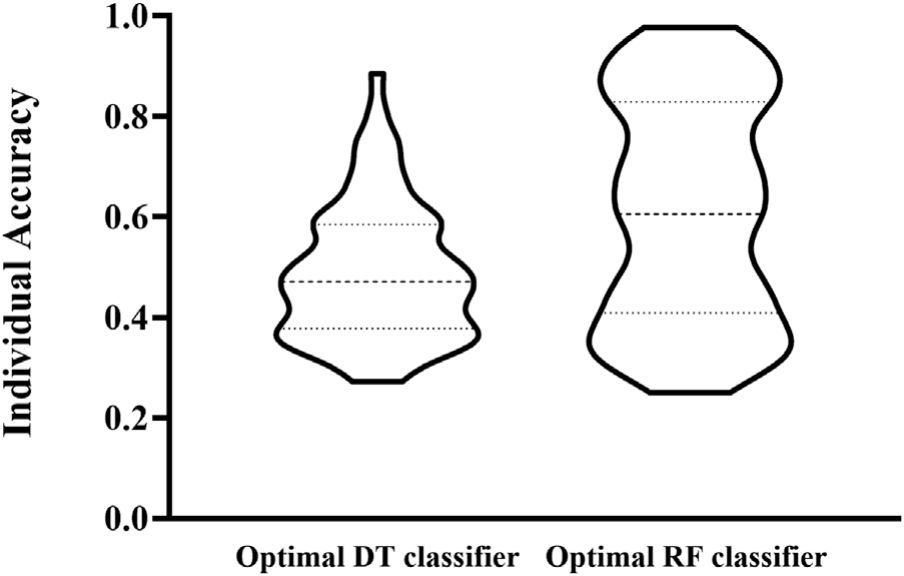
Violin plot to show the performance of two optimal classifiers on all cell types. RF classifier is evidently superior to DT classifier.

**FIGURE 4:**
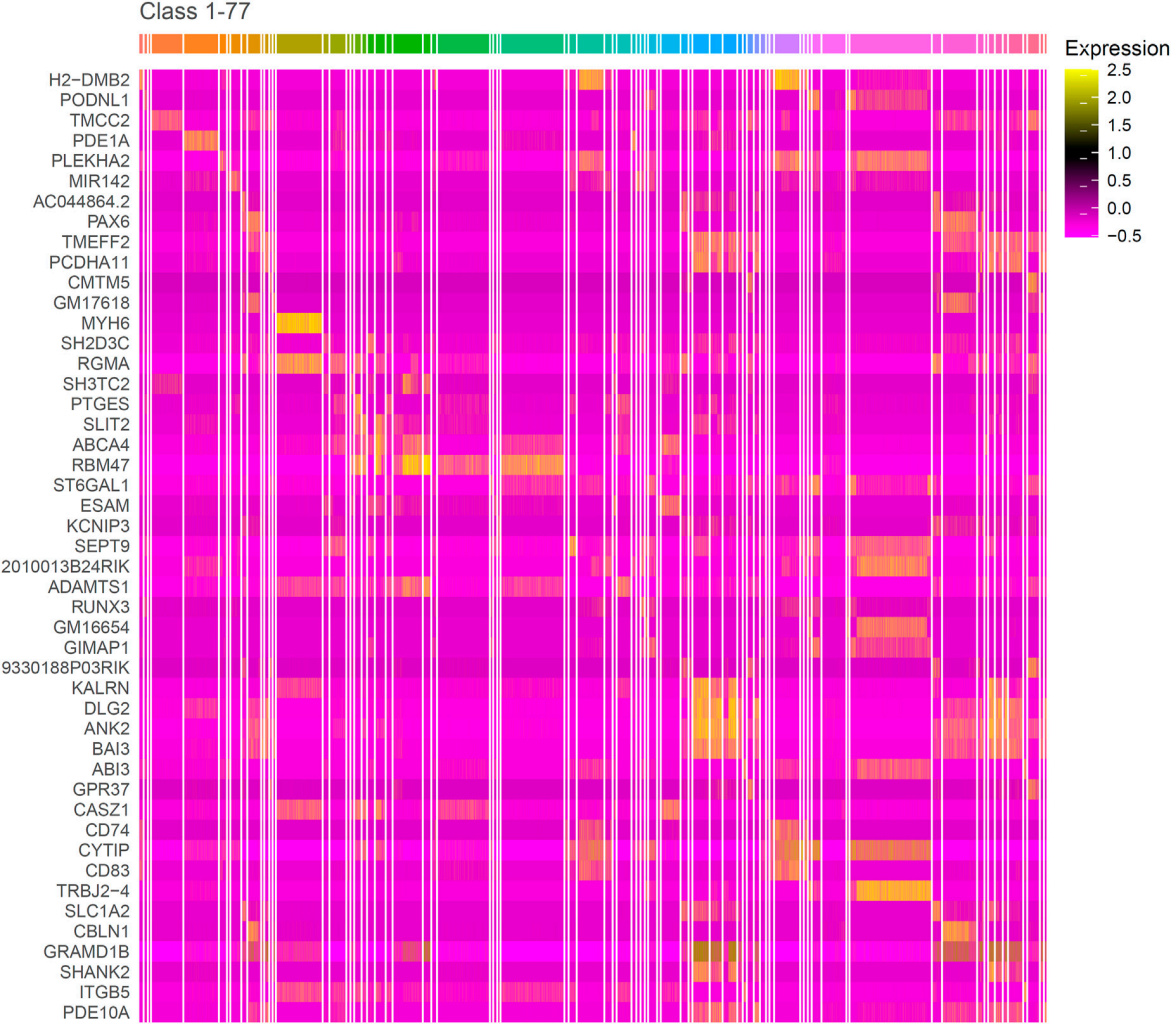
Heatmap of genes in the optimal gene subset obtained by MCFS and IFS. The bright color corresponding to the gene in the heatmap in some classes indicates that this gene is transcriptionally active in these classes. Similarly, if the gene is darker in the class, then it is transcriptionally inactive in this class. The marker genes identified by this research can distinguish different cell types.

The IFS curve indicated that RF had the greatest MCC (0.780) at 445 features. When the top 210 features were used in DT, the greatest MCC were 0.595. Accordingly, the optimal RF and DT classifiers were constructed. The ACC values of these two classifiers were 0.789 and 0.609, respectively, as listed in Table 1. The individual accuracies of them are also shown in Supplementary Table S3, which are illustrated in Figure 3. Evidently, the optimal RF classifier was superior to the optimal DT classifier. For the 445 features used in the optimal RF classifier, we used FindAllMarkers function in Seura package to extract differentially expressed genes for each cell type and adopted logFC to rank these genes in each cell type. The top gene in each cell type was selected, resulting in 73 genes. After excluding genes differentially expressed in more than 1 cell type, 47 genes were obtained. Their expression levels on 77 cell types are illustrated in a heatmap, as shown in Figure 4. It can be observed that some gene features shown good ability to distinguish different mouse cell types and application potential as marker genes for certain cell clusters.

**TABLE 1:** Performance of the key classifiers.

Classification algorithm	Number of features	ACC	MCC
Decision tree	210	0.609	0.595
Random forest	445	0.789	0.780
Random forest	32	0.844	0.838

### 3.3 Classification rules created by the optimal DT classifier

Although the DT classifiers were generally inferior to the RF classifiers, it can provide more medical insights than RF as it is a classic white-box algorithm. Its readability of the working mechanism serves as its strongest distinguishing ability. We could produce a quantitative representation of the features used for different cell type classifications by exploiting the single-tree structure of DT to extract the classification rules.

As the optimal DT classifier adopted the top 210 genes features, all cells were represented by these features. A large tree was learnt from such dataset by DT. 24,257 rules were extracted from this tree, as shown in Supplementary Table S4. Each rule established a limit on the existence of gene features, indicating the relevance of the existence (value >0.5) or non-existence (value ≤0.5) of genes in distinguishing various cell types. Detailed analysis of these rules can be seen in Section 4. However, some rules can distinguish a small number of samples, which is out of the scope of our analysis.

### 3.4 Classification performance optimization using autoencoder and LightGBM

In improving the classification performance, we introduced autoencoder to optimize feature representation. Based on the IFS results, RF achieved the optimal classification performance with MCC of 0.780 when top 445 features were used. These 445 gene features were reconstructed by autoencoder. The reconstructed features were ranked by LightGBM to generate a feature list. Such list was fed into IFS by one-interval step to obtain the optimal feature subsets and optimal RF classifier.

**FIGURE 5:**
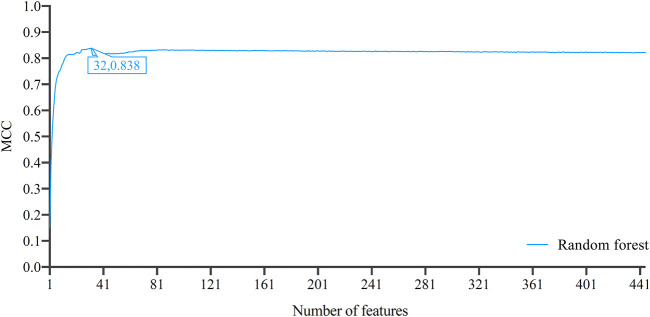
Incremental feature selection (IFS) curves of random forest based on the list by applying LightGBM to the features reconstructed by autoencoder. The optimal MCC of 0.838 is achieved when the number of features is 32, which is better than that based on the original 0–1 representation of genes.

**FIGURE 6:**
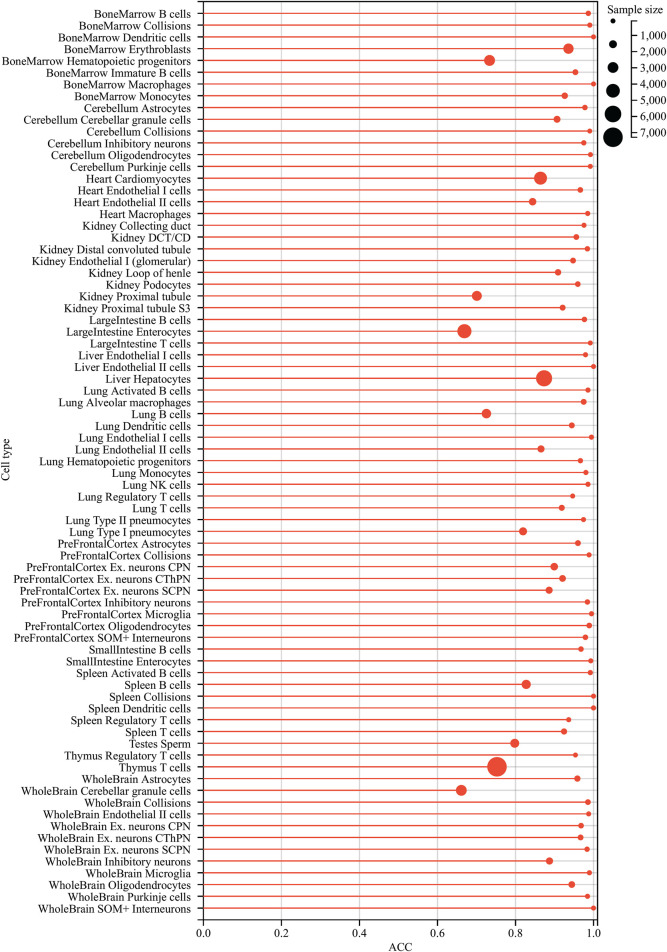
Lollipop plot of individual accuracies yielded by the final random forest classifier for distinguishing different cell types. The circles represent the number of cells contained in different cell types. Some individual accuracies of this classifier optimized by autoencoder can reach up to 1, whereas no individual accuracies are lower than 0.6, indicating the effectiveness of the classifier for cell type classifications.

Similar to Figure 2, the IFS curve was plotted, as shown in Figure 5. The detailed IFS results are shown in Supplementary Table S5. The optimal RF classifier was constructed with MCC of 0.838 using the top 32 features in the feature list. The ACC of this classifier was 0.844 (Table 1). Compared with the previous optimal RF classifier (MCC = 0.780 and ACC = 0.789, see Table 1), the MCC was improved by 0.058 and ACC increased 0.055 after reconstructing features by autoencoder. All individual accuracies of this classifier are shown in Figure 6. All of them were quite high (higher than 0.6). Compared with the performance of the previous optimal RF classifier (Figure 3), they were evidently improved. Such result proved the effectiveness of autoencoder. The final constructed RF classifier can be used for the classification of cells based on single-cell ATAC-seq data.

## 4 Discussion

Our study presented a computational pipeline for analyzing the cell types of mice in single-cell ATAC-seq data. Cells isolated from 13 distinct tissues were further divided into 77 different cell types. By characterizing the chromatin accessibility at single-cell resolution, the status of chromatin accessibility within the gene region was considered as features. They were analyzed by feature selection methods, IFS method and classification algorithms. Lots of essential genes and classification rules were obtained. Here, we focused on some gene features and rules to discuss the relevance of chromatin accessibility in cell type discrimination, which may reveal the important roles of chromatin accessibility in transcriptional regulation and identify cell-type-specific regulatory patterns.

### 4.1 Analysis of chromatin accessibility features by MCFS

The genes were ranked in a list according to the evaluation results of MCFS. Genes with high ranks were more important than others. Here, we selected some top genes for detailed analysis, which are listed in Table 2.

**TABLE 2:** Essential genes related to chromatin accessibility.

Rank in the list	Gene symbol	Description
1	H2-Dmb2	histocompatibility 2, class II, locus Mb2
2	Trbd2	T cell receptor beta, D region 2
4	Trbj2-4	T cell receptor beta joining 2–4
5	Trbj2-1	T cell receptor beta joining 2–1
6	Trbd1	T cell receptor beta, D region 1
7	Trbj2-2	T cell receptor beta joining 2–2
9	Trbj2-3	T cell receptor beta joining 2–3
10	Trbj2-5	T cell receptor beta joining 2–5
11	Trbj2-7	T cell receptor beta joining 2–7
12	Tenm2/Odz2	teneurin transmembrane protein 2

Our analysis identified the chromatin accessibility at the gene region of *H2-Dmb2* to be highly related to the classification of cell types. The protein products encoded by *H2-Dmb2* belong to the MHC class II beta chain paralogues, which are anchored in the membrane, and such products play a central role in peptide binding. MHC class II molecules are specifically expressed in antigen presenting cells such as dendritic cells or macrophages, thereby generating a biased expression of H2-Dmb2 primarily in the spleen, lymph node, and other immune-activated tissues (Rudensky et al., 1991; Cresswell, 1994; Yue et al., 2014). Given the specific expression pattern of H2-Dmb2 across tissues and cells, gene *H2-Dmb2* shows high indicative value for distinguishing antigen-presenting cells and immune-activated tissues; thus, this gene can serve as a biomarker. The ortholog gene of *H2-Dmb2* in human, namely, *HLA-DMB*, plays a critical role in the interaction between antigenic peptides and MHC class II molecules. The aberrant expression of HLA-DMB is associated with many diseases, including diabetes mellitus, autoimmune disease, infection, and cancer (Siegmund et al., 1999; Morel et al., 2004; Callahan et al., 2008; Aissani et al., 2014). Although the detailed mechanisms underlying disease progression remain unknown, the important role of HLA-DMB in antigen presentation cannot be neglected. Understanding the chromatin accessibility in *HLA-DMB* will contribute to revealing the regulatory mechanism and potential targets for disease treatment.

Among the most relevant features identified by our analysis, we found that alterations in chromatin accessibility are associated with many T cell receptor (TCR)-related genes, such as *Trbd1*, *Trbd2*, and *Trbj2*. In a single cell, the TCR beta chain is generated by the somatic recombination of variable V), joining J), diversity D), and constant C) gene segments. The recombination of different segments provides a wide range of antigen recognition for T cell function (Bassing et al., 2002). TCR genes are particularly expressed in T cells; therefore, they display a biased expression pattern in tissues with high infiltration of T lymphocyte. A TCR-β-targeting study by Mathieu et al. demonstrated that chromatin remodeling is associated with the control of TCR gene activation through several epigenetic regulatory mechanisms, and this process can influence the developmental control of TCR gene recombination (Mathieu et al., 2000). This finding indicates the important role of chromatin accessibility in modulating gene expression and consequent function alterations, which provides support for our results, that is, chromatin accessibility of TCR-related genes is highly related to cell functions and cell type classification.

The chromatin accessibility status of gene *Tenm2* (also called *ODZ2*) was identified as another relevant feature for distinguishing cell and tissue types. *Tenm2* is a protein coding gene, which is involved in neural development and cellular signal transduction (Rubin et al., 2002). Given the pivotal role of *Tenm2* in neuronal cells, its transcriptional products exhibit a biased expression primarily in the central nervous system, brain, and other neural-related tissues as demonstrated by Mouse ENCODE transcriptome data (Yue et al., 2014). Although gene *Tenm2* has been reported to be associated with diseases such as periodontitis and anosmia (Alkelai et al., 2016; Sayad et al., 2020), the linkage between *Tenm2* and diseases was primarily built on the basis of genomic studies. Our analysis highlighted the epigenetic modification on gene regulation, indicating that chromatin accessibility at the gene region plays a crucial role in the selective expression of genes, which can serve as cell type-specific markers.

### 4.2 Analysis of decision rules of chromatin accessibility by DT

In improving the explicability of the features implicated in the classification, we performed a quantitative computational analysis using DT. A large number of decision rules involving 210 critical features were built to identify 77 cell types. We focused on the associations between the quantitative features and indicated cell types. Thus, we explored the relevance of the chromatin accessibility tendency of genes in distinguishing cell types through a literature review. Our study provided insights into disentangling cell-type-specific chromatin accessibility and suggested the new epigenetic markers of each cell type.

In the decision rules for identifying the cell type of heart cardiomyocyte, the *Myh6* gene required a relatively high chromatin accessibility, whereas the *Trbv31* and *Nrxn1* genes required low chromatin accessibility. The *Myh6* gene encodes the alpha heavy chain subunit of cardiac myosin, which is the key component of muscle cells. As demonstrated by the Mouse ENCODE transcriptome study, the expression of Myh6 is highly restricted toward heart tissues (Yue et al., 2014). A recent publication proposed that the repressive chromatin assembly on the *Myh6* promoter can silence the expression of Myh6 and impair cardiac contraction (Han et al., 2016). This finding confirmed the crucial role of *Myh6* chromatin modification in cardiac phenotypes, which indicates that the accessible chromatin status of *Myh6* is an essential marker for functional cardiomyocytes. *Trbv31* is a TCR-related gene, and it displays specific expression in T cells (Isobe et al., 1985). The criterion requiring a low chromatin accessibility of *Trbv31* reflects a low gene expression, which is consistent with the actual condition, that is, rare lymphocytes reside within the heart cardiomyocyte environment. *Nrxn1* encodes a single-pass type I membrane protein, which belongs to the neurexin family. Given that neurexins are cell-surface receptors that are restrictedly located at nervous synapses (Südhof, 2008), the Nrxn1 protein is not expressed in heart cardiomyocytes. Therefore, *Nrxn1* serves as a negative marker indicating heart cardiomyocytes.

Among the decision rules for liver hepatocytes, 43 features were involved in the criteria, 42 of which required low chromatin accessibility of genes, whereas only one gene displayed a positive marker, that is, *Slc27a2*. The protein encoded by *Slc27a2* is a fatty-acid coenzyme, which plays a key role in lipid biosynthesis and fatty acid degradation (Steinberg et al., 1999). The biased expression of Slc27a2 in liver and kidney tissues has been demonstrated by a previous study (Yue et al., 2014). The decision rules by our analysis indicate that a high chromatin accessibility of *Slc27a2* is a positive marker indicating liver hepatocytes. The negative features for liver hepatocytes are mostly specific markers of other cell types, such as the aforementioned genes *Trbv31* and *Myh6*, which are specifically expressed in T and cardiac cells, respectively. In addition, another gene (*Lef1*) was identified as a negative marker for liver hepatocytes. This gene encodes a transcription factor that can bind to T-cell receptor enhancer, and it is involved in the Wnt signaling pathway (Petropoulos et al., 2008). A biased expression of Lef1 in the thymus and spleen was demonstrated, which is consistent with its specificity in lymphocytes (Yue et al., 2014). These observations indicated that positive and negative features identified in this analysis contribute to the classification of corresponding cell type.

The relatively high chromatin accessibility of TCR-related genes such as *Trbv31* and *Trbj2* was required to indicate the cell type of thymus T cells. In addition, another positive feature, which is the chromatin accessibility of gene *Lrmp*, was identified to be involved in the decision rules for thymus T cells. *Lrmp*, also known as *Irag2*, encodes a lymphoid-restricted membrane protein, which can regulate the development of lymphoid cell lines (Behrens et al., 1994). RNA profiling data sets generated by the Mouse ENCODE project demonstrated the biased expression of *Lrmp* in thymus tissue (Yue et al., 2014). Our results indicated that in addition to post-transcriptional regulations, modifications of chromatin accessibility play important roles in gene expression control, which can be used as epigenetic markers for distinguishing lymphoid cells.

The decision rules for identifying sperm cells from testes include 45 criteria, among which the high chromatin accessibility of gene *Nol4* was identified by our analysis. *Nol4* is a cancer/testis antigen, and it has been reported to be involved in cancer progression (Kim et al., 2021). Cancer/testis antigens are a group of proteins with normal expression restricted to testicular germ cells but not in adult somatic tissues. In this study, our analysis showed that the chromatin accessibility pattern of the *Nol4* gene was highly related to the classification of testicular sperm cells, presenting a reasonable relevance between the expression of *Nol4* and testicular cells in non-malignant contexts and indicating the potential mechanism of cancer/testis antigen expression through chromatin accessibility modifications.

In this study, a series of quantitative rules was constructed to predict the category of cerebellar granule cells. Among these decision rules, *Cbln1* and *Arpp21* genes required high chromatin accessibility to distinguish cerebellar granule cells. Gene *Cbln1* encodes a cerebellum-specific precursor protein, namely, precerebellin, which is highly enriched in postsynaptic structures of Purkinje cells (Urade et al., 1991). Research by Hirai et al. demonstrated that Cbln1 was secreted from cerebellar granule cells, which have important functions in Purkinje neurons (Hirai et al., 2005). *Arpp21* encodes a cAMP-regulated phosphoprotein, which is enriched in the cerebellar cortex. The high level of Arpp21 mRNA was detected in the cerebellar cortex by *in situ* hybridization and Northern blot analysis (Brene et al., 1994). All these results confirmed the biased expression of Cbln1 and Arpp21 in cerebellum tissues, which support the predictive values of these genes for distinguishing cerebellar granule cells.

## 5 Conclusion

This study computationally investigated the characteristic chromatin accessibility of different mouse cell types at single-cell resolution. The most relevant features and quantitative decision rules were identified through several machine learning algorithms, indicating the potential epigenetic markers for each cell type. Detailed discussion was performed to explore the functional linkage between the chromatin accessibility pattern of genes and the indicated cell types. Many of the identified genes were biased or restrictedly expressed in specific tissues or cells, meaning they can serve as potential biomarkers for the corresponding cell types based on existing experimental evidence and publications. In addition, our study highlighted the epigenetic modification of chromatin in gene expression regulation, implying the critical roles of chromatin accessibility in cell function. Considering the interpretability of features, we primarily focused on features of the chromatin accessibility pattern of genes in cell type discrimination. The classifiers using features reconstructed by autoencoder showed excellent performance. Our study also provides insight into a comprehensive understanding of the genome-wide chromatin accessibility and generic markers in cell lines and tissues.

## Data Availability

Publicly available datasets were analyzed in this study. This data can be found here:https://www.ncbi.nlm.nih.gov/geo/query/acc.cgi?acc=GSE111586.
